# Switch of Intravitreal Therapy for Macular Edema Secondary to Retinal Vein Occlusion from Anti-VEGF to Dexamethasone Implant and Vice Versa

**DOI:** 10.1155/2017/5831682

**Published:** 2017-07-30

**Authors:** Amelie Pielen, Anima Desiree Bühler, Sonja Ute Heinzelmann, Daniel Böhringer, Thomas Ness, Bernd Junker

**Affiliations:** ^1^University Eye Hospital, Hanover Medical School, Hanover, Germany; ^2^Eye Center, University Hospital Freiburg, Freiburg, Germany

## Abstract

**Purpose:**

To evaluate the anatomical and functional outcome of intravitreal dexamethasone implant for macular edema secondary to central (C) or branch (B) retinal vein occlusion (RVO) in patients with persistent macular edema (ME) refractory to intravitreal antivascular endothelial growth factor (VEGF) treatment compared to treatment naïve patients and to dexamethasone-refractory eyes switched to anti-VEGF.

**Methods:**

Retrospective, observational study including 30 eyes previously treated with anti-VEGF (8 CRVO, 22 BRVO, mean age 69 ± 10 yrs), compared to 11 treatment naïve eyes (6 CRVO, 5 BRVO, 73 ± 11 yrs) and compared to dexamethasone nonresponders (2 CRVO, 4 BRVO, 69 ± 12). Outcome parameters were change in best-corrected visual acuity (BCVA) and central foveal thickness (CFT) measured by spectral-domain optical coherence tomography.

**Results:**

Mean BCVA improvement after switch to dexamethasone implant was 4 letters (*p* = 0.08), and treatment naïve eyes gained 10 letters (*p* = 0.66), while we noted no change in eyes after switch to anti-VEGF (*p* = 0.74). Median CFT decrease was most pronounced in treatment naïve patients (−437 *μ*m, *p* = 0.002) compared to anti-VEGF refractory eyes (−170 *μ*m, *p* = 0.003) and dexamethasone-refractory eyes (−157, *p* = 0.31).

**Conclusions:**

Dexamethasone significantly reduced ME secondary to RVO refractory to anti-VEGF. Functional gain was limited compared to treatment naïve eyes, probably due to worse BCVA and CFT at baseline in treatment naïve eyes.

## 1. Introduction

Visual impairment secondary to central or branch retinal vein occlusion (CRVO, BRVO) is mostly caused by macular edema. Intravitreal treatment with either anti-VEGF (vascular endothelial growth factor) or corticosteroids is efficacious and safe [1–3]. Anti-VEGF agents that were currently used are ranibizumab (Lucentis, Novartis Pharma, Switzerland) [4–7], aflibercept (Eylea, Bayer AG, Germany) [8–13], and off-label bevacizumab (Avastin, Roche, Germany) [14, 15]. Among intravitreal corticosteroids, dexamethasone implant (Ozurdex, Allergan, Ireland) is a device approved for macular edema secondary to RVO [16, 17] and diabetes. Pivotal trials that led to approval were conducted in parallel so that head-to-head comparison between anti-VEGF agents and dexamethasone implant was missing until very recently. Consequently, evidenced-based recommendations for treatment of macular edema could only be based on indirect comparison, rendering a decision for the first- and second-line therapeutic treatment recommendation almost impossible [1, 2, 18]. Treatment for macular edema could be initiated with both options and should consider the individual ophthalmological disposition and the patients' circumstances (characteristics to consider are, among others, age, lens status, presence of glaucoma, and mobility).

Head-to-head trials comparing dexamethasone and ranibizumab for macular edema due to RVO are COMO (http://clinicaltrials.gov [19]), COMRADE-B [20], COMRADE-C [21], and COMRADE-Extension trials [submitted by Feltgen et al.]. Results of direct comparison show that both treatments lead to significant improvement of best-corrected visual acuity (BCVA) and macular morphology, but continuous treatment with anti-VEGF ranibizumab given on a pro re nata (PRN) regimen is superior compared to dexamethasone implant at six months (given at a minimum of six months, following the European label and COMRADE trials [20, 21]) as well as compared to PRN dexamethasone after 12 months (given at a minimum of 5-month intervals, COMO trial [19]). Retrospective comparative real-life studies suggest a comparable effect of anti-VEGF injections and dexamethasone implant based on PRN regimen for both [22, 23]. Current experts' consensus recommend intravitreal anti-VEGF first line with a minimum of 3 consecutive monthly injections [3, 24–27]. In case of insufficient effect and persistent or recurrent macular edema, a switch between intravitreal treatments is recommended. This could either be a switch between different anti-VEGF agents or a switch to dexamethasone implant. There is no evidence from prospective randomized controlled trials (RCT) investigating such a switch. But results from pivotal trials as well as knowledge on switch of intravitreal therapy in age-related macular degeneration [28, 29] support the supposed approach.

We conducted the present retrospective observational study to investigate the effects of switch between intravitreal therapy on function and morphology in patients who presented with macular edema secondary to BRVO or CRVO and received either initial anti-VEGF treatment or dexamethasone implant.

## 2. Materials and Methods

This retrospective, observational study was conducted in accordance with the Declaration of Helsinki (1964) and Good Clinical Practice. Before patient recruitment, the study was reviewed and approved by an independent Ethical Committee.

We searched our data for patients with treatment for macular edema secondary to BRVO or CRVO with a switch in intravitreal treatment. Patients had received either (i) anti-VEGF intravitreal injections followed by dexamethasone implant (group: Anti-VEGF_Dexamethasone), (ii) dexamethasone implant followed by anti-VEGF (Dexamethasone_anti-VEGF), or (iii) dexamethasone implant only (treatment naïve group: Dexamethasone). The latter group served as real-life control of treatment effects of dexamethasone implant in RVO. The decision to switch intravitreal therapy was based on clinical findings on examination and could be classified as poor response or no response either in functional (best-corrected visual acuity, subjective visual acuity) or morphological parameters (central foveal thickness (CFT) in spectral-domain optical coherence tomography (SD-OCT)). In the Anti-VEGF_Dexamethasone group, eyes did not respond (sufficiently) to a minimum of three consecutive monthly intravitreal anti-VEGF injections before switch to dexamethasone implant. In the Dexamethasone_anti-VEGF group, eyes did not respond to one or more dexamethasone implants and showed recurrence of macular edema from month two to three onwards after implantation, due to the European label of dexamethasone implant at the time of treatment patients could receive a second implant only after 6 months after the first implant. We recorded the reason for the switch if one of the following classifications was documented as main reason for change of intravitreal therapy: deterioration, stagnation, patient's choice, decompensation of intraocular pressure (IOP), and not known.

Outcome parameters were change in BCVA (logMAR) and CFT (*μ*m) measured by SD-OCT before and after treatment initiation or switch, respectively.

### 2.1. Statistical Analysis

We longitudinally compared visual acuities at baseline (before change of treatment) to the best visual acuities after change and to the visual acuities on record, respectively. We used paired *t*-tests to assess statistical significance. These calculations were performed for all three treatment groups separately. We did not compensate for multiple testing due to the explorative nature of this retrospective project. Central retinal thickness was analyzed alongside visual acuity using analogous calculations.

## 3. Results

The search for patients treated for macular edema secondary to BRVO or CRVO resulted in 47 patients (one eye per patient). Analysis included 30 eyes in the Anti-VEGF_Dexamethasone group (8 CRVO, 22 BRVO, median age 72 years (yrs), mean age 69 ± 10 yrs), compared to 11 treatment naïve eyes (6 CRVO, 5 BRVO, median age 80 yrs, mean age 73 ± 11 yrs) and compared to 6 eyes in the Dexamethasone_anti-VEGF group (2 CRVO, 4 BRVO, median age 69 yrs, mean age 69 ± 12 yrs). The median number of anti-VEGF injections before the switch was 6 (quartiles 3.25; 10), compared to 1.5 dexamethasone implants (quartiles 1; 2) before the switch to anti-VEGF. Patient characteristics and reason for switch are shown in Table 1. The most frequent reasons for a switch were stagnation of BCVA and/or CFT due to macular edema (47% Anti-VEGF_Dexamethasone; 67% Dexamethasone_anti-VEGF) and deterioration (40% and 33%, resp.). Switch to anti-VEGF injections due to IOP increase was only documented for one patient in the Anti-VEGF_Dexamethasone group and none in the Dexamethasone_anti-VEGF group. IOP increase independent of a switch occurred more frequently after intravitreal treatment with dexamethasone implant compared to anti-VEGF. All patients were sufficiently treated with local antiglaucomatous treatment to reduce IOP; none received glaucoma surgery.

### 3.1. Functional and Morphological Results after Switch of Intravitreal Therapy

**Figure 1:**
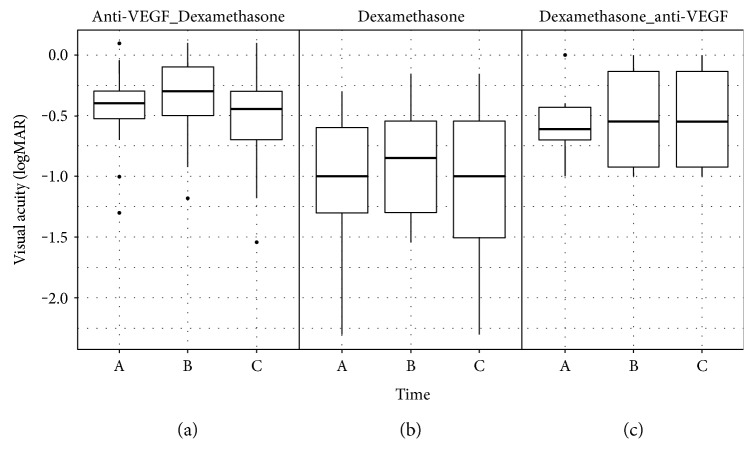
Mean BCVA improvement after dexamethasone implant in anti-VEGF refractory eyes was 4 letters ((a) change in logMAR 0.42 to 0.36, *p* = 0.08); treatment naïve eyes gained 10 letters after dexamethasone implant ((b) change in logMAR 1.07 to 0.82, *p* = 0.66), while we noted no significant change in eyes refractory to dexamethasone implant after switch to anti-VEGF ((c) change in logMAR 0.55 to 0.52, *p* = 0.74). A = BCVA before switch from anti-VEGF to dexamethasone (a) or vice versa (c) or before treatment (b); B = best BCVA after switch/treatment at 88 days [70; 176], 92 days [87; 100], and 123 days [96; 210]; and C = at the end of observation (median follow-up was 4.3 months for the Anti-VEGF_Dexamethasone group, 3.1 months for the Dexamethasone group, and 6.0 months for the Dexamethasone_anti-VEGF group).

**Figure 2:**
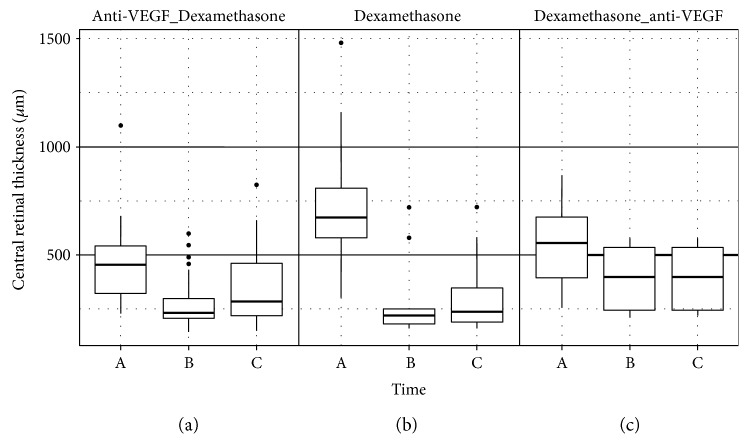
Mean CFT decrease was most pronounced in treatment naïve eyes ((b) from 675 *μ*m [580; 810] to 238 *μ*m [188; 348], change −437 *μ*m, *p* = 0.002) compared to Anti-VEGF_Dexamethasone group ((a) from 455 *μ*m [323; 542] to 285 *μ*m [219; 460], change −170 *μ*m, *p* = 0.003) and Dexamethasone_anti-VEGF treated eyes ((c) from 555 *μ*m [395; 675] to 398 *μ*m [245; 535], change −157 *μ*m, *p* = 0.31). A = CFT before switch from anti-VEGF to dexamethasone (a) or vice versa (c) or before treatment (b); B = CFT at the time of the best BCVA after switch/treatment (at 88 days [70; 176], 92 days [87; 100], and 123 days [96; 210]); and C = at the end of observation (median follow-up was 4.3 months for the Anti-VEGF_Dexamethasone group, 3.1 months for the Dexamethasone group, and 6.0 months for the Dexamethasone_anti-VEGF group).

Switch from anti-VEGF to dexamethasone after a median of 6 anti-VEGF injections led to BCVA improvement of 4 letters (*p* = 0.08, Figure 1()) and decrease of CFT from 455 *μ*m [323; 542] to 285 *μ*m [219; 460] (change −170 *μ*m, *p* = 0.003, Figure 2()). Switch from dexamethasone to anti-VEGF after mean 1.5 implants led to a stabilization of BCVA (*p* = 0.74) despite a change in CFT from 555 *μ*m [395; 675] to 398 *μ*m [245; 535] (change −157 *μ*m, *p* = 0.31). The most pronounced improvement in BCVA and CFT was noted in treatment naïve eyes after dexamethasone implant (BCVA +10 letters, *p* = 0.66; CFT from 675 *μ*m [580; 810] to 238 *μ*m [188; 348], change −437 *μ*m, *p* = 0.002). Mean CFT at the end of follow-up remained significantly reduced in the Anti-VEGF_Dexamethasone group (285 *μ*m [219; 460]) as well as in the Dexamethasone group (238 *μ*m [188; 347]), while we noticed a persistent higher CFT at the end of follow-up in the Dexamethasone_anti-VEGF group (398 *μ*m [245; 535]). Notably, there was a difference in follow-up between groups: Median follow-up was 4.3 months (129 days [75; 335]) for the Anti-VEGF_Dexamethasone group, 3.1 months (94 days [87; 135]) for the Dexamethasone group, and 6.0 months (181 days [156; 261]) for the Dexamethasone_anti-VEGF group.

The rate of “dry eyes” defined as CFT equal or less than 225 *μ*m was 46% (Anti-VEGF_Dexamethasone), 50% (Dexamethasone), and 17% (Dexamethasone_anti-VEGF) at the time of the best BCVA and 29%, 40%, and 17%, respectively, at the end of follow-up.

The time of the best BCVA was 88 days [70; 176], 92 days [87; 100], and 123 days [96; 210] after switch or initiation of therapy, respectively.

Ischemia and subfoveal atrophy of the retinal pigment epithelium were assessed in all SD-OCT scans and fluorescein angiography (if available), but we did not record any in the eyes of all groups.

## 4. Discussion

A positive effect on BCVA and CFT is seen in both of our study groups after switch of intravitreal therapy for macular edema secondary to RVO either from anti-VEGF to dexamethasone implant or vice versa. A comparable positive response to switch from anti-VEGF to dexamethasone was seen in a group of 18 patients, who showed visual improvement of 0.25 logMAR and reduction of macular edema by −146 *μ*m [30]. Another study investigated 48 patients and concluded that switch from anti-VEGF to dexamethasone seemed to be more beneficial in short-term visual acuity and long-term morphological results compared to a switch from dexamethasone to anti-VEGF [31]. But results for the latter group were limited by group size (8 versus 40) comparable to the difference in our study. The positive response seems to apply to switch of therapy in recalcitrant or recurrent macular edema secondary to RVO. In contrast, there seems to be no positive or additive effect if dexamethasone is given after an initial upload of 3 anti-VEGF injection as a fixed combination compared to dexamethasone alone in treatment naïve RVO eyes [32].

In our study, BCVA gain after dexamethasone implant was limited in eyes treated before with median 6 anti-VEGF injections compared to treatment naïve eyes. This effect might be due to worse BCVA and higher CFT in treatment naïve eyes. The rate of CRVO was higher in the Dexamethasone group compared to both switch groups. This could well contribute to the worse baseline BCVA in the treatment naïve eyes as well as the limited BCVA at the end of follow-up. ME following CRVO is more pronounced compared to BRVO, and patients often need more frequently a higher number of intravitreal treatment compared to BRVO. Notably, there was a difference in follow-up between groups which could also contribute to the effects seen (3.1 months dexamethasone versus 4.3 months after switch to dexamethasone and 6.0 months after switch to anti-VEGF). The effect seen in treatment naïve eyes after dexamethasone implant was comparable to results in pivotal dexamethasone trials GENEVA [16, 17] and results of head-to-head trials COMRADE-B [20], COMRADE-C [21], and COMO [19]. After the relevant improvement in BCVA and CFT following dexamethasone implant, we noticed the characteristic decrease of both, BCVA and CFT, at the end of observation in the Dexamethasone group.

Similar but less pronounced effects were visible in the Anti-VEGF_Dexamethasone group. After the switch, we noticed an increase in BCVA, which attenuated until the end of observation. The initial decrease in CFT corresponded well with the improved BCVA, while we did not see a corresponding pronounced increase in CFT at the end of observation. This could be due to limited morphological response in pretreated eyes, which might be more limited the longer or more frequent the previous treatment. Long-term data on anti-VEGF therapy in RVO show that initial BCVA improvement could be stabilized up to 4 years of treatment (RETAIN study [33]). On the other hand, we know that morphological effects appear before functional effects and long-standing macular edema may harm the macula irrevocably, which is a reason to treat macular edema as soon as possible after onset and visual impairment. Results of all pivotal trials on anti-VEGF treatment showed that gain in BCVA was limited initially as well as on the long-term, if treatment was deferred by 6-month sham treatment [4, 5, 8, 11, 14]. There is evidence that early anti-VEGF treatment may reduce the risk and frequency of recurrent macular edema [34].

Results of our group Dexamethasone_anti-VEGF are certainly limited due to the small patient number. Comparable limitations apply to a previous study investigating the switch from anti-VEGF to dexamethasone (40 eyes) and vice versa (8 eyes) [31]. The discrepancy between group size within a real-life setting might be attributable to various reasons including the decision to start more often with anti-VEGF due to preferred practice patterns or possible negative adverse effects of dexamethasone (cataract and IOP), resulting in more eyes in the group of Anti-VEGF_Dexamethasone switch than the other. But results are still valuable, showing that the switch from dexamethasone to anti-VEGF might reduce recalcitrant macular edema (CFT) and stabilize visual acuity. This is the only group in which we noticed no drop in BCVA at the end of observation. Macular edema following RVO needs repetitive intravitreal treatment, and reinjection of anti-VEGF is possible every four weeks allowing for as little morphological and functional fluctuation as possible. On the contrary, dexamethasone implant was approved for use every 6 months. Results of the GENEVA trial [16, 17] as well as COMRADE-B [20], COMRADE-C [21], and COMO [19] show that the effect of dexamethasone implant on BCVA and CFT is most pronounced after 2 months and diminishes from then onwards. Many authors come to the conclusion that a reimplantation is necessary after 3 or 4 months to prevent undulation and stabilize the gain in BCVA.

Current recommendations on treatment of macular edema secondary to RVO recommend initial treatment with multiple anti-VEGF injections as safest option to start with [3, 24, 25, 27]. There is little evidence from head-to-head comparison between different anti-VEGF, but similar effects were shown and it is supposed that the effects of different anti-VEGF agents are noninferior in comparison (MARVEL study [35], SCORE-2 [36]). If macular edema is refractory or recurrent, most experts' consensus recommend a switch within the group of anti-VEGF agents and secondary a switch to intravitreal corticosteroids. Among corticosteroids, dexamethasone implant might be preferred to triamcinolone because of the standardized dosing and less visual disturbance by the implant compared to triamcinolone. Our results add to the knowledge on intravitreal treatment of macular edema due to RVO and support recommendations to switch.

## 5. Conclusions

Intravitreal dexamethasone significantly reduced macular edema due to RVO that was refractory to anti-VEGF intravitreal treatment. However, gain in function as well as morphological improvement were limited after the switch compared to treatment naïve eyes. This could be attributed to significantly worse BCVA and CFT at baseline in treatment naïve macular edema. Switch from dexamethasone to anti-VEGF could stabilize BCVA and CFT. Factors to predict patients' response to anti-VEGF or dexamethasone intravitreal therapy before treatment initiation remain to be determined.

## Figures and Tables

**Table 1:** Patients' baseline characteristics and reason for switch of intravitreal therapy.

	Anti-VEGF to dexamethasone *n* = 30	Dexamethasone to anti-VEGF *n* = 6	Dexamethasone *n* = 11
BRVO	73% (22)	67% (4)	45% (5)
CRVO	20% (6)	17% (1)	55% (6)
Gender (% female)	50%	50%	45%
Age (at baseline [years])	69 ± 10	70 ± 13	74 ± 12
BCVA before switch (mean ± SD [logMAR])	0.42 ± 0.28	0.55 ± 0.34	1.07 ± 0.69
BCVA before switch (mean ± SD [logMAR])	0.36 ± 0.31	0.52 ± 0.45	0.88 ± 0.50
Number of intravitreal injections before switch (median, quartiles)	6 [3.25; 10]	1.5 [1;2]	
Reason for switch			
(i) Deterioration	40% (12)	33% (2)	
(ii) Stagnation	47% (14)	67% (4)	
(iii) Patients' choice	3% (1)	0	
(iv) IOP decompensation	3% (1)	0	
(v) Not known	7% (2)	0	

BRVO/CRVO: branch/central retinal vein occlusion; BCVA: best-corrected visual acuity.
